# Detection of Circulating Tumor Cells (CTCs) in Malignant Pleural Mesothelioma (MPM) with the “Universal” CTC-Chip and An Anti-Podoplanin Antibody NZ-1.2

**DOI:** 10.3390/cells9040888

**Published:** 2020-04-05

**Authors:** Taiji Kuwata, Kazue Yoneda, Masataka Mori, Masatoshi Kanayama, Koji Kuroda, Mika K. Kaneko, Yukinari Kato, Fumihiro Tanaka

**Affiliations:** 1 Second Department of Surgery (Chest Surgery), University of Occupational and Environmental Health, 1-1 Iseigaoka, Yahatanishi-ku, Kitakyushu, Fukuoka 807-8555, Japan; t-kuwata@med.uoeh-u.ac.jp (T.K.); masataka-m@med.uoeh-u.ac.jp (M.M.); masatoshi-kanayama@med.uoeh-u.ac.jp (M.K.); kuroda-k@med.uoeh-u.ac.jp (K.K.); ftanaka@med.uoeh-u.ac.jp (F.T.); 2 Department of Antibody Drug Development, Tohoku University Graduate School of Medicine, 2-1 Seiryo-machi, Aoba-ku, Sendai, Miyagi 980-8575, Japan; k.mika@med.tohoku.ac.jp (M.K.K.); yukinarikato@med.tohoku.ac.jp (Y.K.); 3 New Industry Creation Hatchery Center, Tohoku University, 2-1, Seiryo-machi, Aoba-ku, Sendai, Miyagi 980-8575, Japan

**Keywords:** circulating tumor cells (CTCs), malignant pleural mesothelioma (MPM), podoplanin

## Abstract

Circulating tumor cell (CTC) is a potentially useful surrogate of micro-metastasis, but detection of rare tumor cells contaminated in a vast majority of normal hematologic cells remains technical challenges. To achieve effective detection of a variety of CTCs, we have developed a novel microfluidic system (CTC-chip) in which any antibody to capture CTCs is easily conjugated. In previous studies, we employed an antibody (clone E-1) against podoplanin that was strongly expressed on mesothelioma cells. The CTC-chip coated by the E-1 antibody (E1-chip) provided a modest sensitivity in detection of CTCs in malignant pleural mesothelioma (MPM). Here, to achieve a higher sensitivity, we employed a novel anti-podoplanin antibody (clone NZ-1.2). In an experimental model, MPM cells with high podoplanin expression were effectively captured with the CTC-chip coated by the NZ-1.2 antibody (NZ1.2-chip). Next, we evaluated CTCs in the peripheral blood sampled from 22 MPM patients using the NZ1.2-chip and the E1-chip. One or more CTCs were detected in 15 patients (68.2%) with the NZ1.2-chip, whereas only in 10 patients (45.5%) with the E1-chip. Of noted, in most (92.3%, 12/13) patients with epithelioid MPM subtype, CTCs were positive with the NZ1.2-chip. The CTC-count detected with the NZ1.2-chip was significantly higher than that with the E1-chip (*p* = 0.034). The clinical implications of CTCs detected with the NZ1.2-chip will be examined in a future study.

## 1. Introduction

Circulating tumor cells (CTCs) are tumor cells that shed from solid tumors and circulate in the peripheral blood. As CTCs play a pivotal role in development of distant metastases, CTCs potentially provide important clinical information such as prognosis and therapeutic effect in a variety of malignant tumors [1,2,3,4]. However, detection of rare CTCs contaminated in a large number of normal hematologic cells remains technical challenges. In fact, a variety of systems for detection of CTCs have been developed [5,6,7,8,9], but the CellSearch (Merarini Silicon Biosystems, Huntington Valley, PA, USA) is the only approved system for the clinical use. The CellSearch is an automated system in which CTCs are immunomagnetically isolated with an antibody against epithelial cell adhesion molecule (EpCAM) that is abundantly expressed on epithelial tumor cells [10]. Accordingly, CTCs without EpCAM expression, such as tumor cells undergoing epithelial-mesenchymal transition (EMT) and non-epithelial tumor cells, may not be detected with the CellSearch. In our previous study, we evaluated CTCs using the CellSearch in patients with malignant pleural mesothelioma (MPM). CTCs were detected in only one-third (32.7%) patients, which was mainly caused by negative or low expression of EpCAM on MPM cells [11]. To capture a wide variety of CTCs including tumor cells without EpCAM expression, we have developed a novel microfluidic system (“universal CTC-chip system”) in which any antibody to capture CTCs is easily conjugated [12,13,14,15,16].

Podoplanin, a transmembrane mucin-type sialoglycoprotein, plays an important role in the prenatal development of lymphatic vasculature and lung. In the adulthood, podoplanin is expressed on some normal tissues such as lymphatic vessels and type I alveolar epithelium [17,18]. Podoplanin is also expressed on a wide variety of malignant tumors [19,20,21,22,23,24,25], which may contribute to tumor progression mainly by promoting aggregation and activation of platelet [26,27,28,29,30]. Podoplanin is a well-known diagnostic marker of MPM, as mesothelioma cells show high expression of podoplanis whereas the specific function has yet to be determined [24]. Accordingly, to capture MPM cells, we coated the CTC-chip with an anti-podoplanin antibody. In our previous studies, we used a common anti-podoplanin antibody (clone E-1), and MPM cells were effectively captured with the CTC-chip [14,15,16]. In addition, the CTC-chip provided a superior sensitivity in detection of CTCs in MPM patients over the CellSearch [16]. In the current study, to achieve a higher sensitivity, we employed a novel and highly-reactive anti-podoplanin antibody (clone NZ-1.2 [23]) to detect CTCs in MPM.

## 2. Materials and Methods

### 2.1. Cell Lines

Human epithelioid-subtype MPM cell lines (ACC-MESO-4, ACC-MESO-1, and NCI-H226), biphasic-subtype MPM cells line (MSTO-211H), and sarcomatoid-subtype MPM cell line (NCI-H28) were employed. ACC-MESO-4 and ACC-MESO-1 were purchased from the Riken BioResource Center (Tsukuba, Japan), and the other cell lines were from American Type Culture Collection (ATCC; Manassas, VA, USA). Cells were cultured in RPMI-1640 (FUJIFILM Wako Pure Chemical Corporation, Osaka, Japan) supplemented with 10% fetal bovine serum (Thermo Fisher Scientific Inc., Waltham, MA, USA) at 37 °C and 5% CO_2_.

### 2.2. Flow Cytometry for Evaluation of Podoplanin Expression

Cells were incubated with the anti-podoplanin antibody, NZ-1.2 (50 µg/mL) for 60 min at room temperature. Next, cells were washed with PBS and were incubated with an Alexa Flour 488-conjugated goat anti-rat IgG (H + L) (Thermo Fisher Scientific Inc.) diluted at 1:100. Flow cytometric analysis was performed using the EC800 Cell Analyzer (Sony, Tokyo, Japan) and the FlowJo software (BD Bioscience, Franklin Lakes, NJ, USA). The mean fluorescence intensity (MFI) ratio was determined as the ratio of the mean fluorescein intensity of NZ-1.2 to that of the negative control.

### 2.3. Preparation of CTC-Chip

The CTC-chip was used after a two-step coating, as described previously [14,15,16]. Briefly, the chip was first incubated with a base-antibody at the concentration of 200 µg/mL overnight at 4 °C. Thereafter, the chip was incubated with a capture-antibody (an anti-podoplanin antibody, clone NZ-1.2 or clone E-1 [Santa Cruz Biotechnology, Dallas, TX, USA]) for 60 min at room temperature. A mouse anti-rat IgG antibody (SouthernBiotech, Birmingham, AL, USA) was used as the base antibody when the chip was coated with the NZ-1.2 antibody (“NZ1.2-chip”), and a goat anti-mouse IgG antibody (SouthernBiotech) when the chip was coated with the E-1 antibody (“E1-chip”).

### 2.4. Evaluation of Cell-Capture Efficiency

Cell-capture efficiency was evaluated as described previously [14,15,16]. In brief, 100 tumor cells labeled with CellTrace CSFE Cell Proliferation kit (Thermo Fisher Scientific Inc.) were suspended in 1 mL of the blood sampled from a healthy volunteer (K.T.). The cell suspension sample was applied to the CTC-chip, which was monitored and recorded with a fluorescence microscope (CKX41; Olympus, Tokyo, Japan) and a digital video camera (Sony). The total number of tumor cells applied to the CTC-chip (N-total) was determined as the number of cells that passed through the inlet of the CTC-chip. The number of captured cells (N-captured) was determined as the number of CFSE-labeled cells remained on the CTC-chip. The cell-capture efficiency was represented as N-captured/N-total. Experiments were performed in triplicate.

### 2.5. Clinical Evaluation of CTCs in MPM Patients

The peripheral blood was sampled from patients with pathological diagnosis of MPM, and was drawn to a sample tube (BD Vacutainer EDTA-2K; Becton, Dickinson and Company, Franklin Lakes, NJ, USA). A 1 mL of the blood was taken from the tube and was applied to the NZ1.2-chip, and another 1 mL of the blood taken from the same sample tube was applied to the E1-chip.

Cells captured on the CTC-chip were stained with an anti-cytokeratin (CK) antibody (rabbit polyclonal; Abcam, Cambridge, MA, USA) followed by incubation with an anti-rabbit IgG antibody conjugated Alexa Fluor^®^ 594 (Thermo Fisher Scientific Inc.), an anti-CD45 antibody (rat monoclonal, clone YTH24.5; Abcam) followed by incubation with an anti-rat IgG antibody conjugated Alexa Fluor^®^ 488 (Thermo Fisher Scientific Inc.), and with Hoechst33342 (Cell Signaling Technology, Danvers, MA, USA). Each cell with round to oval morphology, a Hoechst33342-positive nucleus, positive staining for CK in the cytoplasm, and negative staining for CD45 was judged as a CTC [16].

The revised TNM criteria were used to determine clinical stage (c-stage) [16]. This study was approved by the Ethics Committee of Medical Research, University of Occupational and Environmental Health, Japan (H26-15), and was performed in accordance with the Declaration of Helsinki.

### 2.6. Statistical Analysis

The proportions of un-paired categorical data were compared by the chi-square test or the Fisher’s exact test, and those of paired categorical data were compared by the McNemar’s test. Continuous data were compared with a non-parametric test (Wilcoxon signed rank test for paired data, and Mann-Whitney U-test or Kruskal-Wallis test for un-paired data). Differences were considered to be statistically signified for *p*-value < 0.05. All statistical analysis was performed with GraphPad PRISM software (GraphPad Software, Inc., La Jolla, CA, USA). 

## 3. Results

### 3.1. Podoplanin Expression in MPM Cell Lines

**Figure 1:**
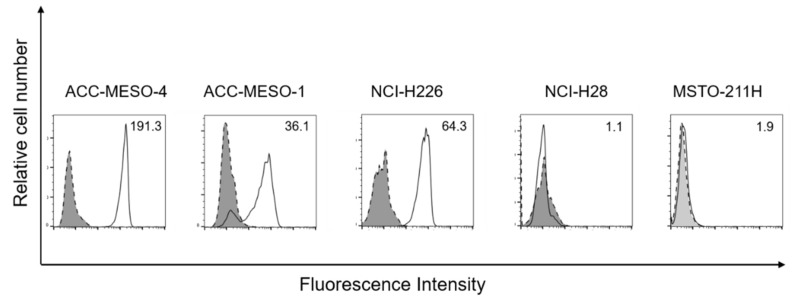
Flow cytometric analysis of podoplanin expression on malignant mesothelioma (MPM) cell lines (ACC-MESO-1, ACC-MESO-4, NCI-H226, NCI-H28, and MSTO-211H). The number in the top right corner in each figure is the mean fluorescein intensity (MFI) ratio.

All epithelioid MPM cell lines (ACC-MESO-1, ACC-MESO-4 and NCI-H226) showed high podoplanin expression. In contrast, non-epithelioid MPM cell lines (NCI-H28 and MSTO-211H) showed no significant podoplanin expression (Figure 1).

### 3.2. Cell-Capture Efficiency of NZ1.2-Chip

**Figure 2:**
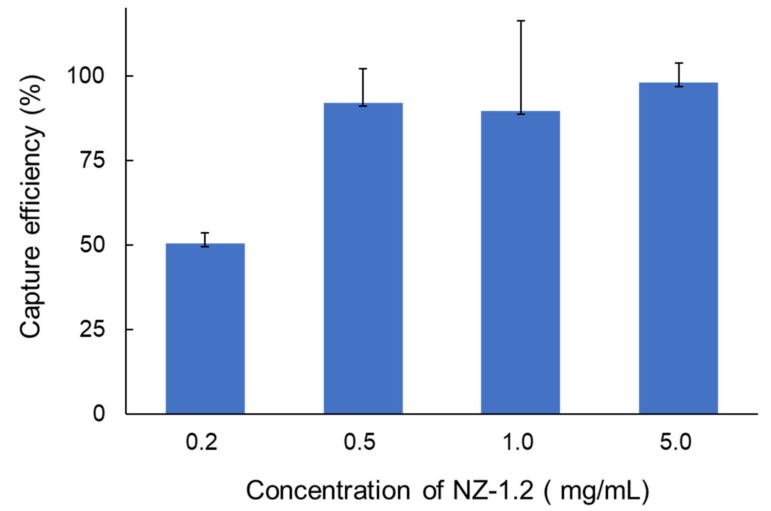
Cell-capture efficiency of the CTC-chip coated with an anti-podoplanin antibody (NZ-1.2) at various concentrations in capturing malignant pleural mesothelioma (MPM) cells (ACC-MESO-4).

First, we examined the optimal concentration of the NZ-1.2 antibody for the CTC-chip using the ACC-MESO-4 cells with the highest podoplanin expression. The highest capture efficiency was achieved at the concentration of 5.0 mg/mL (Figure 2), which was adopted as the optimal concentration of the NZ-1.2 in further experiments.

**Figure 3:**
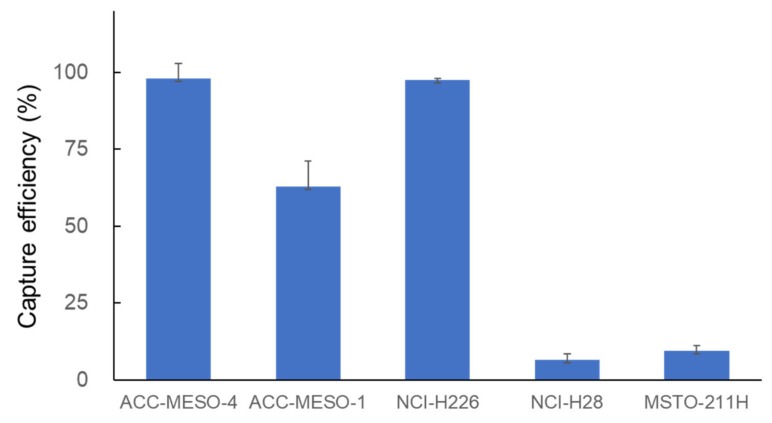
Cell-capture efficiency of the CTC-chip coated with an anti-podoplanin antibody (NZ-1.2) at the concentration of 5 mg/mL in capturing various malignant pleural mesothelioma (MPM) cells (ACC-MESO-1, NCI-H226, NCI-H28, and MSTO-211H).

Next, we examined the efficiency in capturing various MPM cell lines with the NZ1.2-chip. Epithelioid MPM cell lines (ACC-MESO-1 and NCI-H226) with high podoplanin expression were effectively captured, but the cell-capture efficiency for non-epithelioid cell lines (NCI-H28 and MSTO-211H) was low (Figure 3).

### 3.3. Clinical Evaluation of CTCs in MPM Patients

**Figure 4:**
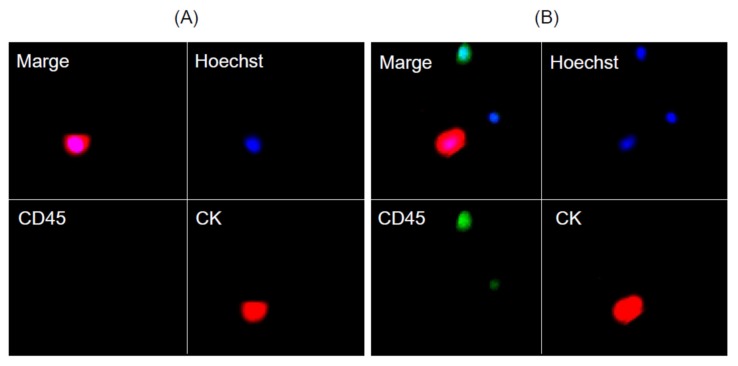
Detection of tumor cells (ACC-MESO-4 cells spiked in blood (**A**) and circulating tumor cells in patients with malignant pleural mesothelioma (**B**)) captured on the CTC-chip coated with an anti-podoplanin antibody (clone NZ-1.2).

We sampled the peripheral blood from a total of 22 MPM patients treated between November 2016 and September 2018 (Table 1) for detection of CTCs (Figure 4) at the diagnosis of MPM. One or more CTCs were detected with either E1-chip or NZ1.2-chip in 19 (86.4%) patients; 6 (27.3%) patients were positive with E1-chip and positive with NZ1.2-chip, 4 (18.2%) patients were positive with E1-chip and negative with NZ1.2-chip, 9 (40.9%) patients were negative with E1-chip and positive with NZ1.2-chip, and 3 (13.6%) patients were negative with E1-chip and negative with NZ1.2-chip. In total, CTCs were detected in 10 (45.5%) patients with the E1-chip and in 15 (68.2%) patients with the NZ1.2-chip. Of noted, in most (12/13, 92.3%) epithelioid MPM patients, CTCs were detected in the peripheral blood with the NZ1.2-chip (Table 1).

**Figure 5:**
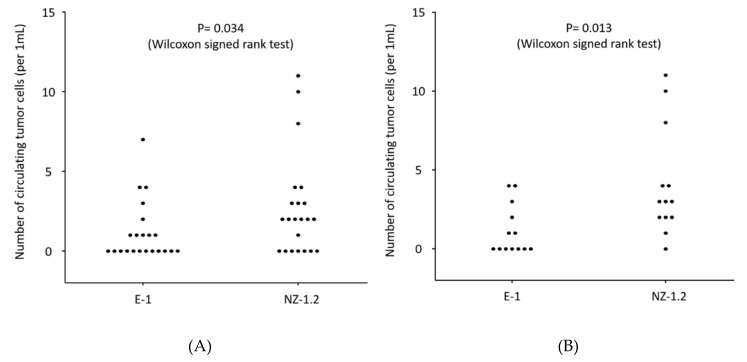

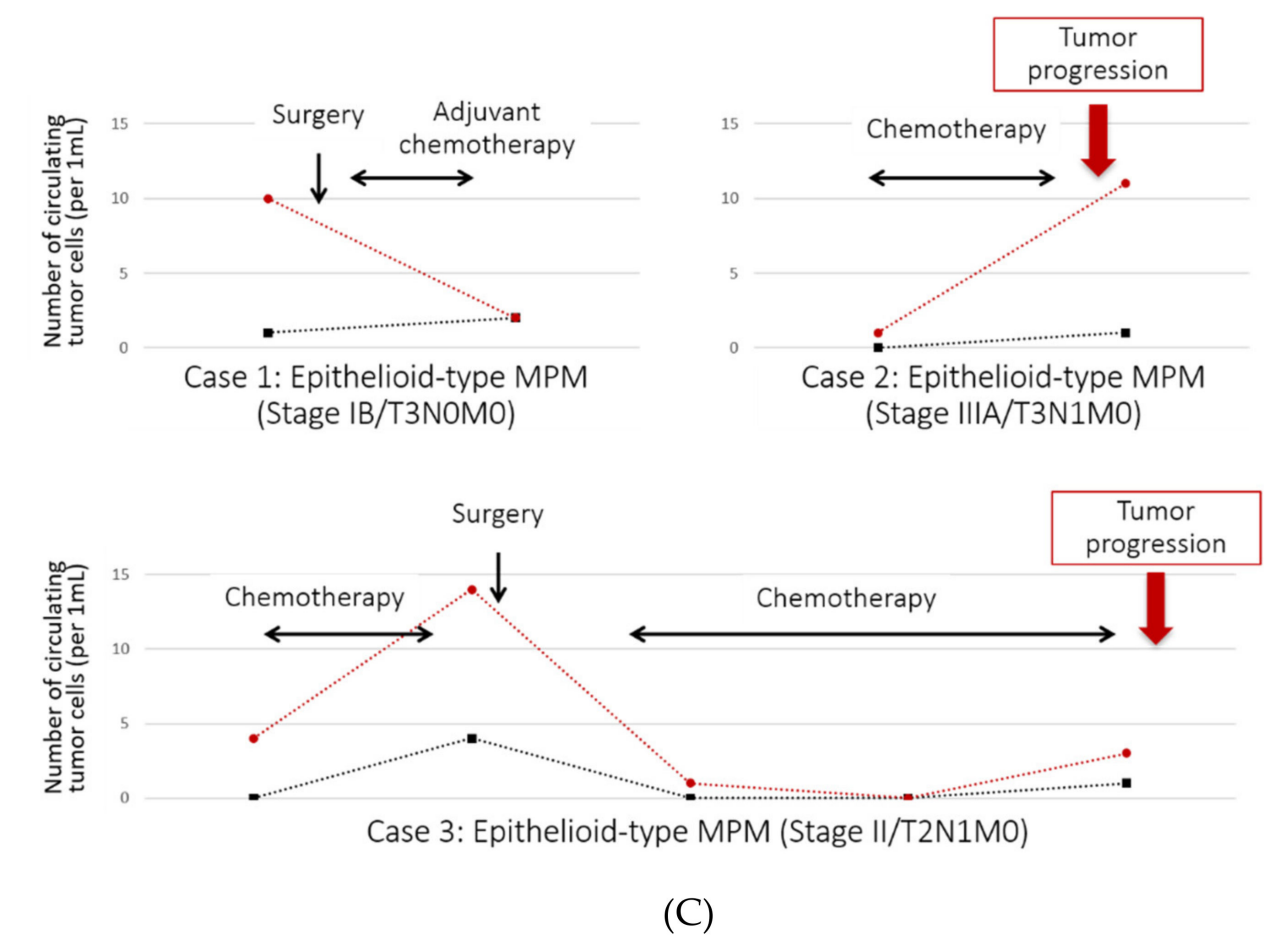
(**A**) The number of circulating tumor cells (CTC-count) detected with the CTC-chip in 1 mL of the peripheral blood sampled from 22 patients with malignant pleural mesothelioma (MPM). The CTC-chip was coated with an anti-podoplanin antibody, clone E-1 (E1-chip) or clone NZ-1.2 (NZ1.2-chip), for capturing tumor cells. A 1 mL of the peripheral blood taken from the same sample tube was applied to the E1-chip or the NZ1.2-chip. (**B**) The number of circulating tumor cells (CTC-count) detected with the CTC-chip in 1 mL of the peripheral blood sampled from 13 patients with epithelioid-type malignant pleural mesothelioma (MPM). (**C**) Changes in the number of circulating tumor cells (CTC-count) detected with the E1-chip (black lines and dots) or the NZ1.2-chip (red lines and dots) during treatment in some selected patients with malignant pleural mesothelioma (MPM).

The distribution of CTC-count detected with the NZ1.2-chip and that with the E1-chip in each sample was shown in Figure 5A. The median CTC-count for the E1-chip and for the NZ1.2-chip were “0” (range, 0–7) and “2” (range, 0–11), respectively, indicating a significantly higher CTC-count was achieved with the NZ1.2-chip (*p* = 0.034). The difference in the CTC-count detected with the E1-chip and the NZ1.2-chip was highly significant in epithelioid patients (median CTC-count, 0 and 3, respectively; *p* = 0.013) (Figure 5B). In some patients, changes in the CTC-count during treatment were monitored with the E1-chip and the NZ1.2-chip (Figure 5C). At most points of time, a higher number of CTCs were detected with the NZ1.2-chip.

**Figure 6:**
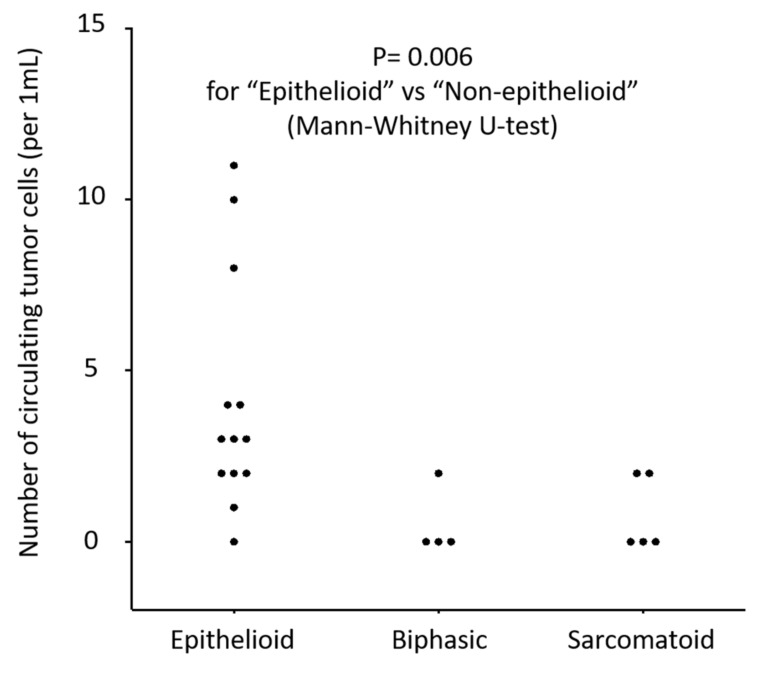
Distribution of the number of circulating tumor cells (CTC-count) detected with the CTC-chip in 1 mL of peripheral blood sampled from patients with malignant pleural mesothelioma (MPM) according to histologic subtype. The CTC-chip was coated with an anti-podoplanin antibody (clone NZ-1.2) to capture CTCs.

**Figure 7:**
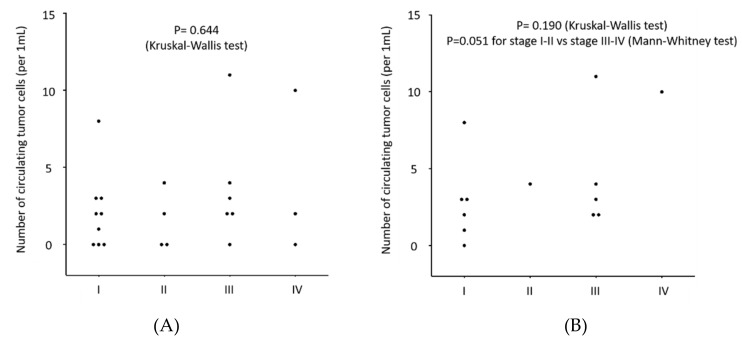
(**A**) Distribution of the number of circulating tumor cells (CTC-count) detected with the CTC-chip in 1 mL of peripheral blood sampled form patients with malignant pleural mesothelioma (MPM) according to clinical stage. The CTC-chip was coated with an anti-podoplanin antibody (clone NZ-1.2) to capture CTCs. (**B**) Distribution of the number of circulating tumor cells (CTC-count) detected with the CTC-chip in 1 mL of peripheral blood sampled form patients with epithelioid malignant pleural mesothelioma (MPM) according to clinical stage.

The median CTC-count detected with the NZ1.2-chip was 3 for epithelioid subtype and 0 for biphasic or sarcomatoid subtype, and the CTC-count was significantly higher in epithelioid MPM patients than in non-epithelioid patients (Figure 6). The CTC-count was not significantly different according to clinical stage (Figure 7A), but the CTC-count trended higher in advanced stage (stage III and IV) among epithelioid patients (*p* = 0.051) (Figure 7B).

## 4. Discussion

In this study, we employed a novel antibody (NZ-1.2) with a higher affinity to podoplanin in capturing MPM cells using the CTC-chip. A number of pathological studies have demonstrated that podoplanin expression is positive in most cases (80–100%) of epithelioid subtype of MPM [31,32]. In the current study, epithelioid-type MPM cell lines showed high podoplanin expression (Figure 1), and were effectively captured on the CTC-chip coated with the NZ-1.2 antibody (cell-capture efficiency, 62.8% for ACC-MESO-1, 97.9% for ACC-MESO-4, and 97.6% for NCI-H226) (Figure 3). In previous studies, the cell-capture efficiency achieved by the CTC-chip coated with a common anti-podoplanin antibody (E-1) was 84.1% for ACC-MESO-4 and 76.3% for NCI-H226 [16]. Whereas direct comparison of cell-capture efficiency may not be feasible, MPM cells were more effectively captured on the CTC-chip when coated with the novel anti-podoplanin antibody (NZ-1.2). Podoplanin may be also expressed on sarcomatoid subtype of MPM, but the positivity seems lower (30–75%) as compared with epithelioid MPM [31,33,34]. In fact, non-epithelioid MPM cell lines (NCI-H28, and MSTO-211H) showed no significant podoplanin, which were not effectively captured on the NZ1.2-chip (Figure 1; Figure 3).

Next, we showed that the NZ1.2-chip enabled sensitive detection of CTCs in the peripheral blood of MPM patients. As MPM cells may not express strong EpCAM expression, CTCs cannot be effectively captured using EpCAM-dependent methods. In fact, the CellSearch provided only a modest sensitivity in detection of CTCs (32.7% [11] and 44.45% [35]). As an EpCAM-independent detection method, Bobek and coworkers employed a size-based preparation method [36]. They reported a promising sensitivity (80%, 4/5) in detection of CTCs in MPM patients, but no further study has been reported. The sensitivity obtained with the NZ1.2-chip (68.2% for all MPM patients and 93.2% for epithelioid patients) in this study might be promising. In addition, the CTC-count detected with the NZ1.2-chip was significantly higher than that with the E1-chip, especially for epithelioid patients (Figure 5A,B). In addition, dynamic changes in the CTC-count evaluated with the NZ1.2-chip during treatment were documented in some patients (Figure 5C). These results may indicate that the NZ1.2-chip provides useful clinical information in the diagnosis and decision-making of treatment for patients with epithelioid sub-type of MPM.

There are several limitations in this study. First, the number of patients included in this retrospective study was only 22, which was too small to draw any definitive conclusion, and we are aware that promising results of the CTC-test in the current study are preliminary. We are now sampling the peripheral blood from newly-diagnosed MPM patients for the CTC-test, which will be presented in the future. In addition, the CTC-test was performed only once for a single blood sample in the current study. The reproducibility of the CTC-test should be investigated using multiple samples obtained from each patient in a future study. To validate the clinical significance of the CTC-test, a multi-institutional prospective study shall be conducted. Second, the clinical impact of the CTC-test should be examined after a longer follow-up time. To reveal the prognostic and predictive value of the CTC-count, we are now conducting retrospective and prospective studies of monitoring the CTC-count in correlation with patient characteristics and clinical course of MPM patients. Third, the NZ1.2-chip may enable a sensitive detection of CTCs in epithelioid MPM patients, but not in non-epithelioid MPM patients with the sensitivity of 33.3%. In this study, non-epithelioid MPM cells (H28 and 211H) without significant podoplanin expression were not effectively captured with the NZ1.2-chip. To overcome the limitation, we are now testing the CTC-chip coated with multiple antibodies such as anti-EGFR antibody in combination with an anti-podoplanin antibody, as EGFR is highly overexpressed on MPM including non-epithelioid subtypes [37,38]. Finally, non-malignant cells such as reactive mesothelial cells may highly express podoplanin, and can be detected in the peripheral blood. In the current study, we did not validate whether cells captured on the NZ1.2-chip were true MPM cells. We have just developed immunostaining of cells captured on the CTC-chip, and will examine loss of nuclear BAP1 expression, which is a useful marker in discrimination between MPM cells and non-malignant mesothelial cells [39].

In conclusion, the NZ1.2-chip achieved a higher performance in capturing CTCs in MPM. Future studies may reveal clinical relevance of CTCs detected with the NZ1.2-chip.

## Figures and Tables

**Table 1:** Characteristics of patients and detection of CTCs with the CTC-chip.

		Number of Patients (Percentage)	Number of CTC-Positive (CTC-Count ≥ 1/mL) Patients (Percentage)	
			E1-Chip	NZ1.2-Chip
Age	Median (range)	68.5 years (54–79)		
Sex	Male	21 (95.5%)	10 (47.6%)	14 (66.7%)
	Female	1 (4.5%)	0 (0%)	1 (100%)
Histologic subtype	Epithelioid	13 (59.1%)	6 (46.2%)	12 (92.3%)
	Biphasic	4 (18.2%)	2 (50.0%)	1 (25.0%)
	Sarcomatoid	5 (22.7%)	2 (40.0%)	2 (40.0%)
Clinical stage	I	10 (45.5%)	3 (30.0%)	7 (70.0%)
	II	4 (18.2%)	3 (75.0%)	2 (50.0%)
	III	5 (22.7%)	3 (60.0%)	4 (80.0%)
	IV	3 (13.6%)	1 (33.3%)	2 (66.7%)
Total of patients		22 (100.0%)	10 (45.5%)	15 (68.2%)
